# Prevalence of antibiotic use in a tertiary care hospital in Italy, 2008–2016

**DOI:** 10.1186/s13052-019-0645-7

**Published:** 2019-05-20

**Authors:** Marta Luisa Ciofi degli Atti, Carmen D’Amore, Jacopo Ceradini, Valerio Paolini, Gaetano Ciliento, Giuseppe Chessa, Massimiliano Raponi

**Affiliations:** 1 Unit of Clinical Epidemiology, Bambino Gesù Children’s Hospital, Piazza di Sant’Onofrio 4, 00165 Rome, Italy; 2 Medical Direction, Bambino Gesù Children’s Hospital, Piazza di Sant’Onofrio 4, 00165 Rome, Italy

**Keywords:** Antibiotic use, Prevalence, Children, Trend

## Abstract

**Background:**

Few data are available about temporal trends of antibiotic use in hospitalized children. The aim of the current study was to investigate the pattern and trends of antibiotic use over the years 2008–2016 in the largest children’s hospital in Italy.

**Methods:**

Annual point prevalence surveys of antibiotic use were conducted by reviewing medical charts of 0–17 year-old children hospitalized for ≥48 h. Prevalence of antibiotic use was computed by year, type of ward and indication. Trends in prevalence over time were evaluated using the Cochrane-Armitage test. Possibile determinants of antibiotic use were assessed at univariate analysis and through a logistic regression model.

**Results:**

Out of 3015 children, 1516 (50.3%) received antibiotics, 58.1% of which for medical/surgical prophylaxis. Prevalence of antibiotic use increased from 42.0% in 2008 to 56.2% in 2016 (*p* = 0.001). The prevalence of patients receiving antibiotics for medical prophylaxis increased from 6.1% in 2008 to 24.2% in 2016 (*p* < 0.001), whereas the prevalence of patients receiving antibiotics for surgical prophylaxis significantly decreased (from 13.7 to 11.8%; *p* = 0.04); no significant temporal trends were found in antibiotic use for treating infections.

The administration of third-generation cephalosporins for surgical and medical prophylaxis significantly decreased over time, while the proportion of antibiotics prescribed to treat infections after microbiological investigations significantly increased. Year (ORadj: 1.8 in 2016 compared to 2008, *p* < 0.001), age (ORadj ≥1.5 in children ≥1 year, compared to infants ≤2 months, *p* < 0.001), length of stay (LOS) (OR_adj_: 1.4 in case of LOS between 8 and 30 days compared to LOS ≤ 7 days, *p* < 0.001), and type of ward (ORadj: ≥1.3 in intensive-care, surgical and medical-subspecialty units compared to medical units, *p* < 0.001) were significantly and independently associated with antibiotic use.

**Conclusions:**

Comparing prevalence rates of antibiotic use among hospitals and over time should consider differences in patient characteristics, such as age, ward of hospitalization and length of stay. Over the years, we documented an improvement in the choice of antibiotics prescribed for medical and surgical prophylaxis. However, further efforts are needed to avoid antibiotic misuse for medical prophylaxis, and to reduce the empirical use of broad spectrum antibiotics.

**Electronic supplementary material:**

The online version of this article (10.1186/s13052-019-0645-7) contains supplementary material, which is available to authorized users.

## Background

Antibiotics are among the drugs most commonly prescribed to children, and are often used to treat common conditions generally caused by viral agents, against which antibiotics are mostly ineffective [1]. In outpatient settings, nearly 50% of prescriptions of antibiotics to children have been estimated to be unnecessary [2]. In inpatients, overuse of broad spectrum antibiotics and excessively prolonged surgical antibiotic prophylaxis have been frequently reported in hospitalized neonates and children [3, 4].

A judicious use of antibiotics is essential to slow the emergence of antibiotic resistance in bacteria, and to extend the useful lifetime of effective antibiotics [5]. Increased microbial resistance to antibiotics is a global public health challenge, which causes severe infections, increased mortality, longer hospital stays and higher costs [6, 7]. Antibiotic overprescribing is also associated with an increased risk of adverse effects [8], including more frequent re-attendance and increased medicalization of self-limiting conditions [9].

There is a wide variability in antibiotic consumption between countries, and Italy is one of the european nations with the highest use of antibiotics, both in outpatient and inpatient settings [10]. In 2007–2008, the annual rate of outpatient antibiotic prescriptions to Italian children and adolescents was estimated around 1.0 prescriptions/person/year, being by far the highest compared to the other european countries [11]. As regards hospital antibiotic consumption, the estimated prevalence of antibiotic use in pediatric wards in Italy in 2011 was significantly higher than the mean European estimate, being 51.5% (95% CI: 44.0–59.0%) versus 35.4% (95% CI, 33.6–37.2%) [12], respectively.

Few data are available about temporal trends of antibiotic use in hospitalized children [13]. The aim of the current study was to investigate the pattern and trends of antibiotic consumption over the period 2008–2016 in the largest children’s hospital in Italy.

## Methods

### Study setting

This study was conducted at the Bambino Gesù Children’s Hospital (Ospedale Pediatrico Bambino Gesù, OPBG), a 607-bed tertiary care academic hospital in the Lazio Region, Italy. Due to a shift towards day-hospital and day-surgery care, the annual hospital inpatient admissions decreased from 33,132 in 2008 to 26,947 in 2016. Over this period, the average diagnosis-related group (DRG) weight - a measure of disease severity - increased from 0.86 in 2008 to 1.04 in 2016. The average length of stay (LOS) increased from 5.4 in 2008, to 6.5 in 2016. Starting 2008, a series of actions have been undertaken to promote the appropriate use of antibiotics, namely: a) dissemination of results of annual prevalence surveys on antibiotic use by posting reports on the hospital intranet website, presenting data in hospital meetings, and discussing actions to be undertaken within the hospital infection control team (from 2008); b) production and dissemination of hospital guidelines on antibiotic surgical prophylaxis (from 2009), c) production and dissemination of hospital guidelines on antibiotic medical prophylaxis (from 2011); d) restriction of use of third-generation cephalosporins for surgical prophylaxis (from 2012).

### Study design and data collection and inclusion criteria

We have conducted annual point prevalence surveys of hospital antibiotic use in years 2008–2016. The surveys were carried out during a maximum of two calendar weeks in the summer period and involved all patients aged 0–17 years hospitalized for at least 48 h. Data were collected from patients’ paper medical charts. Information collected for each patient included: age, sex, length of hospital stay, and ward type. In case of antibiotic prescriptions, we collected information on antibiotic type according to the Anatomical Therapeutic Chemical Classification code, ATC J01) [14], drug brand name, and indication for antibiotic administration, defined as community-acquired infections (CAIs), hospital-acquired infections (HAIs), medical or surgical prophylaxis. For antibiotics used for infections, collected information included whether microbiological investigations were performed prior to starting antibiotic therapy. All data were analyzed anonymously.

### Statistical analysis

Patients were characterized according to demographic factors (age and sex), length of stay and ward of hospitalization. Wards were categorized as medical (including general neonatal and pediatric wards), surgical (e.g., neonatal surgery, pediatric surgery, including subspecialties such as orthopedics, neurosurgery, ear-nose-throat), medical subspecialty (e.g., cardiology, oncology and hematology, bronchopneumology, neonatal semi-intensive care unit), and intensive care units (ICUs including pediatric intensive care unit, neonatal intensive care unit, cardiac intensive care unit).

Prevalence of antibiotic use was the main measure of outcome. It was calculated as a ratio between the number of patients under treatment with at least one antibiotic on the day of the survey, and the total amount of patients hospitalized for at least 48 h. Prevalence of antibiotic use was computed by year, type of ward and indication. Antibiotic classes were stratified by indication of antibiotic prescription.

Trends in patient characteristics and in prevalence of antibiotic use over time were evaluated using the Cochrane-Armitage test. Year, sex, age, length of stay and ward of hospitalization were investigated as possible determinants of antibiotic use. We evaluated the association between all these variables and exposure to antibiotics at univariate analysis and through a logistic regression model. Crude and adjusted odds ratios (ORs), with 95% confidence intervals (CI) were estimated.

All statistical analyses were conducted using STATA 13 (Stata Corporation, College Station, Texas, USA).

## Results

### Prevalence of antibiotic use

A total of 3015 pediatric inpatients were involved in point prevalence surveys conducted between 2008 and 2016. The demographic characteristics of patients remained quite stable over the years (Table 1), while the length of stay and the distribution by type of ward significantly varied over time. In fact, the proportion of children with a length of hospitalization between 8 and 30 days increased from 25.3% in 2008 to 36.5% in 2016 (*p* = 0.02); the proportion of inpatients in surgical wards decreased from 35.9 to 20.2% (*p* = 0.0001) whereas those hospitalized in Medical subspecialty unit increased from 21.1% in 2008 to 29.6% in 2016 (*p* = 0.01).

**Table 1:** Characteristics of patients included in point prevalence surveys by year; OPBG, 2008–2016

	2008 (*N* = 379)	2009 (*N* = 267)	2010 (*N* = 320)	2011 (N = 320)	2012 (*N* = 353)	2013 (*N* = 344)	2014 (*N* = 333)	2015 (*N* = 368)	2016 (*N* = 331)	Total (*n* = 3015)	*P-value for trend*
	N (%)	N (%)	N (%)	N (%)	N (%)	N (%)	N (%)	N (%)	N (%)	N (%)	
Sex											
Female	160 (42.2)	111 (41.6)	148 (46.2)	158 (49.4)	154 (43.6)	143 (41.6)	154 (46.2)	165 (44.8)	159 (48.0)	1352 (44.8)	0.2
Age											
≤2 months	59 (15.6)	44 (16.5)	59 (18.4)	53 (16.6)	66 (18.7)	45 (13.1)	55 (16.5)	63 (17.1)	63 (19.0)	507 (16.8)	0.6
3–11 months	58 (15.3)	42 (15.7)	57 (17.8)	57 (17.8)	64 (18.1)	57 (16.6)	55 (16.5)	54 (14.7)	53 (16.0)	497 (16.5)	0.7
1–5 years	102 (26.9)	77 (28.8)	71 (22.2)	77 (24.1)	92 (26.1)	82 (23.8)	76 (22.8)	98 (26.6)	85 (25.7)	760 (25.2)	0.6
6–11 years	104 (27.4)	62 (23.2)	91 (28.4)	82 (25.6)	88 (24.9)	94 (27.3)	92 (27.6)	84 (22.8)	79 (23.9)	776 (25.7)	0.3
≥ 12 years	55 (14.5)	40 (15.0)	40 (12.5)	51 (15.9)	42 (11.9)	66 (19.2)	55 (16.5)	69 (18.8)	51 (15.4)	469 (15.6)	0.09
Missing	1 (0.3)	2 (0.7)	2 (0.6)	0 (0.0)	1 (0.3)	0 (0.0)	0 (0.0)	0 (0.0)	0 (0.0)	6 (0.2)	
Length of hospital stay (days)											
≤7	220 (58.1)	132 (49.4)	159 (49.7)	165 (51.6)	152 (43.1)	170 (49.4)	150 (45.1)	198 (53.8)	134 (40.5)	1480 (49.1)	0.0008
8–30	96 (25.3)	82 (30.7)	109 (34.1)	95 (29.7)	132 (37.4)	103 (29.9)	111 (33.3)	113 (30.7)	121 (36.5)	962 (31.9)	0.02
> 30	63 (16.6)	52 (19.5)	52 (16.2)	60 (18.7)	69 (19.5)	70 (20.3)	72 (21.6)	57 (15.5)	76 (23.0)	571 (18.9)	0.1
Missing	0 (0.0)	1 (0.4)	0 (0.0)	0 (0.0)	0 (0.0)	1 (0.3)	0 (0.0)	0 (0.0)	0 (0.0)	2 (0.1)	
Ward Type											
Medical unit	131 (34.6)	99 (37.1)	103 (32.2)	117 (36.5)	107 (30.3)	111 (32.2)	126 (37.8)	112 (30.4)	125 (37.8)	1031 (34.2)	0.9
Surgical unit	136 (35.9)	67 (25.1)	93 (29.1)	84 (26.3)	100 (28.4)	88 (25.6)	71 (21.4)	104 (28.3)	67 (20.2)	810 (26.9)	0.0001
Medical subspecialty unit	80 (21.1)	76 (28.4)	91 (28.4)	84 (26.3)	113 (32.0)	112 (32.6)	101 (30.3)	106 (28.8)	98 (29.6)	861 (28.6)	0.01
Intensive care unit	32 (8.4)	25 (9.4)	33 (10.3)	35 (10.9)	33 (9.3)	33 (9.6)	35 (10.5)	46 (12.5)	41 (12.4)	313 (10.4)	0.05

The overall nine-year prevalence of antibiotic use was 50.3% (*n* = 1516). Among the hospital wards, intensive care units had the highest prevalence of antibiotic use, namely 62.0% (194/313 patients admitted in ICUs), followed by 52.1% in medical subspecialty units (449/861), 50.6% in surgical units (410/810), and 44.9% in medical units (463/1031; *p* < 0.001).

Prophylaxis was the main indication for antibiotic use. Children receiving antibiotic for medical or surgical prophylaxis accounted for 34.0% (*n* = 515) and 24.1% (*n* = 366) respectively of all children receiving antibiotics. The proportion of patients who received antibiotics for treating CAIs or HAIs was equal to 24.9% (*n* = 378) and 21.5% (*n* = 326), respectively.

### Trend and determinants of antibiotic use

Trend analysis showed an increase in the prevalence of antibiotic use, from 42.0% in 2008 to 56.2% in 2016 (*p* < 0.001, Table 2). The prevalence of patients treated for medical prophylaxis increased from 6.1% in 2008 to 24.2% in 2016 (p < 0.001), whereas the prevalence of patients treated for surgical prophylaxis significantly decreased (from 13.7 to 11.8%; *p* = 0.04); no significant trends were found in antibiotic use for treating infections.

**Table 2:** Trends in prevalence of antibiotic use by year and indication; OPBG, 2008–2016

	2008 (*N* = 379)	2009 (*N* = 267)	2010 (*N* = 320)	2011 (*N* = 320)	2012 (*N* = 353)	2013 (*N* = 344)	2014 (*N* = 333)	2015 (*N* = 368)	2016 (*N* = 331)	Total (*N* = 3015)	*P-value for trend*
	N (%)	N (%)	N (%)	N (%)	N (%)	N (%)	N (%)	N (%)	N (%)	N (%)	
Patients with antibiotic	159 (42.0)	123 (46.1)	170 (53.1)	160 (50.0)	190 (53.8)	174 (50.6)	165 (49.5)	189 (51.4)	186 (56.2)	1516 (50.3)	0.001
Indication for antibiotic use											
CAIs^a^	50 (13.2)	30 (11.2)	40 (12.5)	54 (16.1)	49 (13.9)	41 (11.9)	28 (8.4)	45 (12.2)	41 (12.4)	378 (12.5)	0.3
HAIs^b^	37 (9.8)	35 (13.1)	39 (12.2)	42 (13.1)	36 (10.2)	28 (8.1)	38 (11.4)	33 (9.0)	38 (11.5)	326 (10.8)	0.4
Surgical prophylaxis	52 (13.7)	35 (13.1)	52 (16.3)	30 (9.4)	46 (13.0)	47 (13.7)	26 (7.8)	39 (10.6)	39 (11.8)	366 (12.1)	0.04
Medical prophylaxis	23 (6.1)	26 (9.7)	56 (17.5)	39 (12.2)	65 (18.4)	61 (17.7)	81 (24.3)	84 (22.8)	80 (24.2)	515 (17.1)	< 0.001

^a^*CAIs* Community-acquired infections, ^b^*HAIs* Hospital-acquired infections

When analyzing the prevalence of antibiotic use by indication and type of ward, we found that the rate of patients in surgical units who received antibiotic for surgical prophylaxis remained stable over time (from 31.6% in 2008 to 35.8% in 2016), while the rate of patients in medical subspecialty units who received antibiotic for medical prophylaxis significantly increased, from 13.8% in 2008 to 46.9% in 2016 (*p* < 0.001). Oncological patients accounted for 31.2% of patients treated with antibiotics in medical subspecialty units; this percentage increased from 18.8% in 2008 to 38.8% in 2016 (*p* < 0.001).

The year was significantly and independently associated with antibiotic use (Table 3). Other independent predictors of antibiotic use were type of ward (ORadj: ≥1.3 in patients admitted in intensive-care, surgical and medical-subspecialty units compared to patients in medical units; *p* < 0.01), age (OR_adj_: ≥ 1.5 in children ≥1 year of age, compared to infants ≤2 months; *p* < 0.001), and length of stay (OR_adj_: 1.4 in case of LOS between 8 and 30 days compared to LOS ≤ 7 days; *p* < 0.001).

**Table 3:** Factors associated with antibiotic use: results of univariate and multivariate logistic analysis

	N. total patients	N. patients with at least 1 prescription	%	Unadjusted OR (CI 95%)	*P*-value	Adjusted OR^a^ (CI 95%)	*P*-value
Year							
2008	379	159	42.0	*1*		*1*	
2009	267	123	46.1	1.2 (0.9–1.6)	0.3	1.2 (0.9–1.6)	0.3
2010	320	170	53.1	1.6 (1.2–2.1)	0.003	1.5 (1.1–2.1)	0.005
2011	320	160	50.0	1.4 (1.0–1.9)	0.03	1.4 (1.0–1.9)	0.03
2012	353	190	53.8	1.6 (1.2–2.2)	0.001	1.6 (1.2–2.1)	0.002
2013	344	174	50.6	1.4 (1.1–1.9)	0.02	1.4 (1.0–1.8)	0.03
2014	333	165	49.5	1.4 (1.0–1.8)	0.04	1.3 (1.0–1.8)	0.05
2015	368	189	51.4	1.5 (1.1–2.0)	0.01	1.4 (1.0–1.9)	0.02
2016	331	186	56.2	1.8 (1.3–2.4)	< 0.001	1.8 (1.3–2.4)	< 0.001
Sex							
Female	1352	656	48.5	*1*		*1*	
Male	1663	860	51.7	1.1 (0.9–1.3)	0.08	1.1 (1.0–1.3)	0.06
Age							
≤2 months	507	233	45.9	*1*		*1*	
3–11 months	497	218	43.9	0.9 (0.7–1.2)	0.5	1.0 (0.8–1.3)	0.8
1–5 years	760	386	50.8	1.2 (1.0–1.5)	0.09	1.5 (1.2–1.9)	0.001
6–11 years	776	425	54.8	1.4 (1.1–1.8)	0.002	1.7 (1.4–2.2)	< 0.001
≥ 12 years	469	250	53.3	1.3 (1.0–1.7)	0.02	1.6 (1.3–2.1)	< 0.001
Missing	6	4	–	–		–	
Length of hospital stay (days)							
≤7	1480	696	47.0	*1*		*1*	
08–30	962	540	56.1	1.4 (1.2–1.7)	< 0.001	1.4 (1.2–1.7)	< 0.001
> 30	571	279	48.9	1.1 (0.9–1.3)	0.5	1.0 (0.8–1.2)	1.0
Missing	2	1	–	–		–	
Ward							
Medical	1031	463	44.9	*1*		*1*	
Surgical	810	410	50.6	1.3 (1.0–1.5)	0.02	1.3 (1.1–1.6)	0.01
Medical subspecialty unit	861	449	52.1	1.3 (1.1–1.6)	0.002	1.3 (1.1–1.6)	0.003
Intensive care	313	194	62.0	2.0 (1.5–2.6)	< 0.001	2.4 (1.8–3.2)	< 0.001

^a^ORs were adjusted for sex, year, age, length of hospital stay, ward type

### Type of molecules

**Fig. 1:**
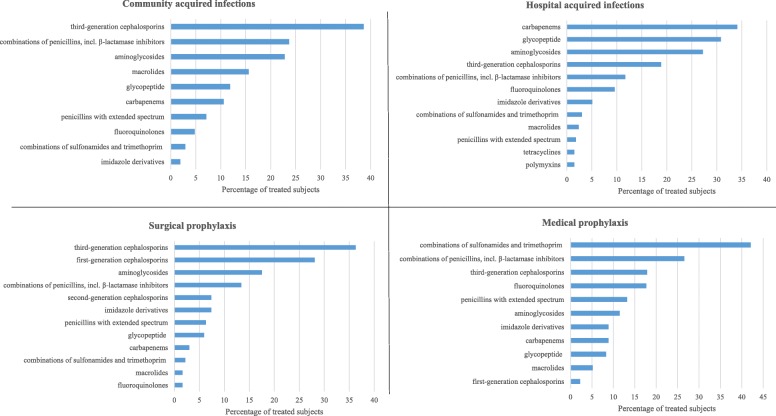
Distribution of antibiotic use, by indication and type of molecules; OPBG, 2008–2016

Throughout the study period, third-generation cephalosporins ranked first both for the treatment of CAIs (where they were administered to 38.6% of patients), and for surgical prophylaxis (36.3%; Fig. 1). Carbapenems represented the most used molecules in HAIs (34.9%), followed by glycopeptide (30.8%) and aminoglicosides (27.2%). Combinations of sulfonamides and trimethoprim were mainly used in medical prophylaxis (41.2%, Fig. 1).

**Fig. 2:**
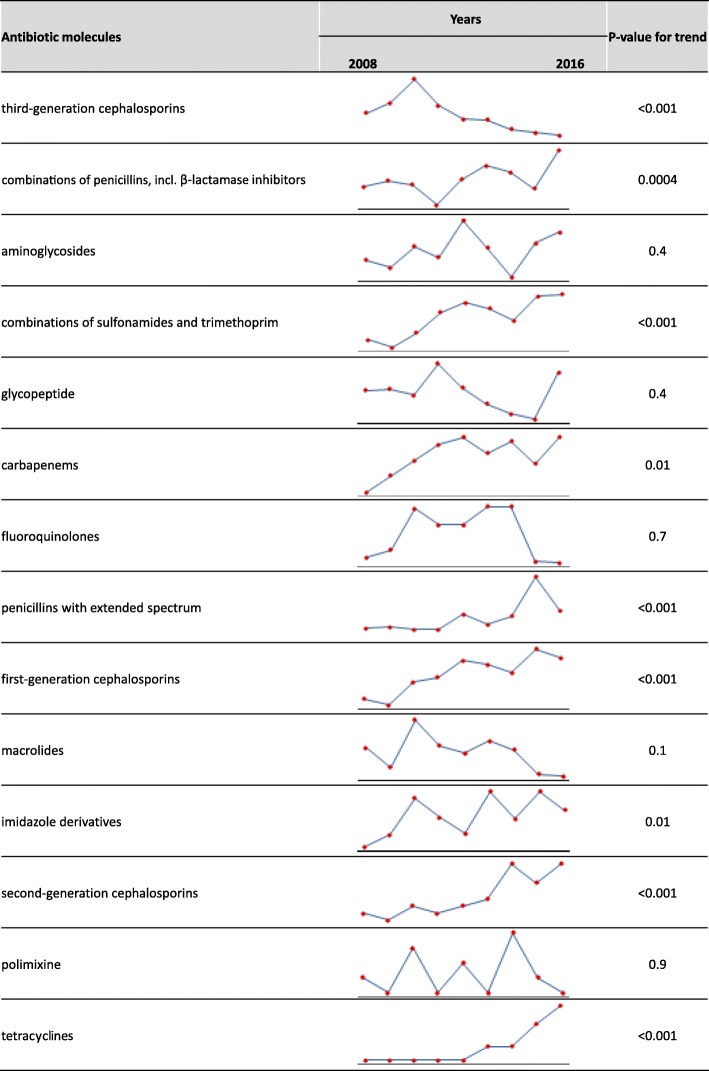
Prevalence of antibiotic use by year and type of molecules; OPBG, 2008–2016

During the study period, a significant increase was observed in the use of combinations of penicillins, incl. β-lactamase inhibitors, first- and second-generation cephalosporins, combinations of sulfonamides and trimethoprim, penicillins with extended spectrum and carbapenems; the use of third-generation cephalosporins significantly decreased over time (Fig. 2).

The use of combinations of penicillins, incl. β-lactamase inhibitors and combinations of sulfonamides and trimethoprim increased mainly in medical prophylaxis (from 13.0 and 43.5% in 2008, to 43.8 and 48.8% in 2016; *p* < 0.05) (see Additional file 1), whereas the use of first- and second-generation cephalosporins increased in surgical prophylaxis, from 5.8 and 1.9% in 2008 to 46.2 and 20.5% in 2016 (*p* < 0.001). The decrease in the administration of third-generation cephalosporins concerned both surgical (from 53.8% in 2008 to 12.8% in 2016, *p* < 0.001) and medical prophylaxis (from 17.4% in 2008 to 6.3% in 2016, *p* < 0.001).

A reduction in glicopeptyde use (from 10.0% in 2008 to 4.9% in 2016, *p* = 0.01) and an increase in penicillins with extended spectrum (from 4.0 to 12.2%, *p* = 0.02) were found in patients treated for CAIs. Concerning HAIs, increasing trends in the use of imidazole derivatives and tetracyclines were observed (from 0% in 2008 to 2.6 and 7.9% respectively in 2016, *p* < 0.05).

Overall, a microbiological test was conducted in 636 (61.2%) treatments out of 1039 prescriptions for infections. This proportion went from 42.3% in 2008 (52/123) to 59.6% in 2016 (68/114, *p* < 0.05). The proportion of treatments prescribed after microbiological testing was significantly higher for antibiotics prescribed for HAIs (73.4%, 367/500), compared to CAIs (49.9%, 269/539; *p* < 0.001).

## Discussion

In this study, antibiotic use significantly increased over time, from 42.0% in 2008 to 56.2% in 2016. These rates are higher than those reported in a recent worldwide point prevalence survey on antibiotic prescriptions in hospitalized children, which estimated a 36.7% global prevalence of use [15]. Comparing crude prevalence rates among hospitals and over time is difficult, because of the differences in patient case-mix, type of hospital, and indication to prescription. In this regard, our patient population was characterized by a high proportion of children admitted in subspecialty medical units (28.6%), and intensive care units (10.4%), where antibiotic use was significantly higher than in surgical and medical units, consistently with the findings of other studies [5, 15, 16].

In this study, medical prophylaxis was the main indication for antibiotic use, with an increasing trend over time: it referred mainly to children admitted in medical subspecialty units, whose proportion increased by 42% from 2008 to 2016, and especially in oncology and hematology. Combinations of sulfonamides and trimethoprim (e.g. cotrimoxazole) and combinations of penicillins, incl. β-lactamase inhibitors resulted to be the antibiotic classes mainly used for medical prophylaxis, being widely used to prevent opportunistic infections in neutropenic patients [17]. However, there is still a percentage of inappropriate use of antibiotics in medical prophylaxis; in fact, about 18% of patients receiving this prophylaxis were administered third-generation cephalosporins, and 9% of them received carbapenems. Although these broad spectrum molecules were mainly prescribed in patients affected by a wide variety of conditions (e.g. cancer, cystic fibrosis, transplants, primary immunodeficiency, asplenia, and preterm infants) [18–23] and requiring aggressive antibiotic treatment considering their immunological status and predisposition to severe infections, further efforts are needed to avoid antibiotic misuse for medical prophylaxis in children, to clearly identify patients who need it, and to define the best antibiotic choice for the different indications.

The rate of inpatient children in surgical wards, who received surgical antibiotic prophylaxis remained stable over the years, however there was a significant improvement in the quality of molecules used. In fact, third-generation cephalosporins were the first-choice for surgical prophylaxis until 2012, when they were replaced by first- or second-generation cephalosporins, as recommended [24, 25]. This result could be due to actions undertaken since 2009 to promote the appropriateness of surgical prophylaxis, including the implementation of hospital guidelines and the restriction of third-generation cephalosporins use in surgical theatres.

For hospitalized patients who have suspected infections, international guidelines reccomend to collect microbiological samples before prescribing an antimicrobial [26]. Premature initiation of antimicrobial therapy, in fact, can suppress bacterial growth and preclude the opportunity to establish a microbiological diagnosis, useful to set a targeted antibiotic therapy [27]. When considering antibiotic therapeutic prescriptions, we noticed that the proportion of therapies administered after microbiological testing significantly increased over time (from 42.3 to 59.6%), and was significantly higher for HAIs (73.4%) compared to CAIs (49.9%). In our study, the use of third-generation cephalosporins as first-choice treatment for CAIs, as well as of carbapenems for HAIs, may be explained within the context of a country, such as Italy, with a high prevalence of antimicrobial resistance [28].

Point prevalence surveys have been used to assess antibiotic use in children [12, 15, 16, 29, 30], but this is the first study investigating the change in prevalence of antibiotic use covering such a long period and taking into account the differences in antibiotic prescription practices for the different therapeutic indications. The main strength of this study is that we conducted the annual point prevalence surveys in the same period of the year, with a standardized methodology. Several epidemiologic studies on antibiotic use have been conducted using a wide range of methods including the defined daily doses (DDDs) [10, 11, 13, 31–34]. However, DDDs are normally assigned based on the use in adults and are not appropriate to monitor antibiotic consumption in children [14]. Point prevalence surveys are simple and feasible, and allow to collect a number of information to assess prescriptions, such as the indication and choice of molecules. For this reason, the conduct of antibiotic point prevalence surveys should be a standard component of antimicrobial stewardship programs targeting children.

The results of this study should be interpreted in the light of some limitations. It was conducted in the largest tertiary care children’s hospital in Italy, where a series of actions were implemented to promote over the years a judicious use of antibiotics. Thus, we cannot assume that its results are representative of the in-hospital trend of antibiotic use in other centers. Information on the beginning of antibiotic therapy was not collected, so we cannot exclude the possibility that some children with CAIs were already under therapy when admitted, which limits the possibility of targeting the drug choice on microbiological results.

## Conclusions

Comparing prevalence rates of antibiotic use among hospitals and over time should consider differences in patient characteristics, such as age, ward of hospitalization and length of hospitalization. We documented an improvement in the choice of antibiotics prescribed for medical and surgical prophylaxis. However, further efforts are needed to avoid antibiotic misuse for medical prophylaxis, and to reduce the empirical use of broad spectrum antibiotics.

## Additional file

Additional file 1: Trend analysis of the proportion of patients treated with antibiotics by indication and type of molecules (panel A: Community-acquired infections, panel B: Hospital-acquired infections, panel C: Surgical prophylaxis and panel D: Medical prophylaxis). (DOCX 36 kb)

## Abbreviations

ATC: Anatomical Therapeutic Chemical Classification code
CAI: Community-acquired infection
DDD: Defined daily dose
DRG: Diagnosis-related group
HAI: Hospital-acquired infection
ICU: Intensive care unit
LOS: Length of stay
OPBG: Ospedale Pediatrico Bambino Gesù
