# Supplementary material for: Prevalence of antibiotic use in a tertiary care hospital in Italy, 2008–2016
Source: Ital J Pediatr. 2019 May 20;45:63. doi: 10.1186/s13052-019-0645-7 (PMC6528368; doi:10.1186/s13052-019-0645-7)
Supplement: Supplementary file 1 — Trend analysis of the proportion of patients treated with antibiotics by indication and type of molecules (panel A: Community-acquired infections, panel B: Hospital-acquired infections, panel C: Surgical prophylaxis and panel D: Medical prophylaxis). (DOCX 36 kb) [file 13052_2019_645_MOESM1_ESM.docx]

**Additional file**

Trend analysis of the proportion of patients treated with antibiotics by indication and type of molecules (panel A: Community-acquired infections, panel B: Hospital-acquired infections, panel C: Surgical prophylaxis and panel D: Medical prophylaxis)

A)

| **Type of molecules** | **2008 (N=50)** | **%** | **2009 (N=30)** | **%** | **2010 (N=40)** | **%** | **2011 (N=54)** | **%** | **2012 (N=49)** | **%** | **2013 (N=41)** | **%** | **2014 (N=28)** | **%** | **2015 (N=45)** | **%** | **2016 (N=41)** | **%** | **Total (N=378)** | **%** | **P-value for**  **trend** |
| --- | --- | --- | --- | --- | --- | --- | --- | --- | --- | --- | --- | --- | --- | --- | --- | --- | --- | --- | --- | --- | --- |
| third-generation cephalosporins | 18 | 36.0 | 11 | 36.7 | 19 | 47.5 | 23 | 42.6 | 13 | 26.5 | 20 | 48.8 | 11 | 39.3 | 17 | 37.8 | 14 | 34.1 | 146 | 38.6 | 0.8 |
| penicillins and β-lactams inhibitors | 17 | 34.0 | 7 | 23.3 | 10 | 25.0 | 4 | 7.4 | 9 | 18.4 | 10 | 24.4 | 10 | 35.7 | 11 | 24.4 | 12 | 29.3 | 90 | 23.8 | 0.9 |
| aminoglycosides | 12 | 24.0 | 3 | 10.0 | 12 | 30.0 | 8 | 14.8 | 12 | 24.5 | 10 | 24.4 | 6 | 21.4 | 13 | 28.9 | 10 | 24.4 | 86 | 22.8 | 0.4 |
| macrolides | 9 | 18.0 | 4 | 13.3 | 7 | 17.5 | 10 | 18.5 | 6 | 12.2 | 8 | 19.5 | 5 | 17.9 | 4 | 8.9 | 6 | 14.6 | 59 | 15.6 | 0.5 |
| glycopeptide | 5 | 10.0 | 6 | 20.0 | 6 | 15.0 | 11 | 20.4 | 9 | 18.4 | 4 | 9.8 | 0 | 0.0 | 2 | 4.4 | 2 | 4.9 | 45 | 11.9 | 0.01 |
| carbapenems | 4 | 8.0 | 3 | 10.0 | 2 | 5.0 | 8 | 14.8 | 7 | 14.3 | 5 | 12.2 | 3 | 10.7 | 4 | 8.9 | 4 | 9.8 | 40 | 10.6 | 0.7 |
| penicillins with extended spectrum | 2 | 4.0 | 0 | 0.0 | 4 | 10.0 | 2 | 3.7 | 5 | 10.2 | 0 | 0.0 | 1 | 3.6 | 8 | 17.8 | 5 | 12.2 | 27 | 7.1 | 0.02 |
| fluoroquinolones | 2 | 4.0 | 2 | 6.7 | 1 | 2.5 | 6 | 11.1 | 2 | 4.1 | 1 | 2.4 | 2 | 7.1 | 2 | 4.4 | 0 | 0.0 | 18 | 4.8 | 0.4 |
| combinations of sulfonamides and trimethoprim | 2 | 4.0 | 1 | 3.3 | 1 | 2.5 | 2 | 3.7 | 1 | 2.0 | 0 | 0.0 | 0 | 0.0 | 2 | 4.4 | 2 | 4.9 | 11 | 2.9 | 0.9 |
| imidazole derivatives | 0 | 0.0 | 1 | 3.3 | 0 | 0.0 | 2 | 3.7 | 0 | 0.0 | 0 | 0.0 | 0 | 0.0 | 3 | 6.7 | 1 | 2.4 | 7 | 1.9 | 0.2 |
| second-generation cephalosporins | 0 | 0.0 | 0 | 0.0 | 0 | 0.0 | 0 | 0.0 | 0 | 0.0 | 0 | 0.0 | 1 | 3.6 | 1 | 2.2 | 0 | 0.0 | 2 | 0.5 | 0.2 |
| tetraciclyne | 0 | 0.0 | 0 | 0.0 | 0 | 0.0 | 0 | 0.0 | 0 | 0.0 | 1 | 2.4 | 0 | 0.0 | 1 | 2.2 | 0 | 0.0 | 2 | 0.5 | 0.3 |
| other antibiotics | 0 | 0.0 | 1 | 3.3 | 0 | 0.0 | 0 | 0.0 | 0 | 0.0 | 0 | 0.0 | 0 | 0.0 | 0 | 0.0 | 0 | 0.0 | 1 | 0.3 | 0.3 |
| first-generation cephalosporins | 0 | 0.0 | 0 | 0.0 | 0 | 0.0 | 0 | 0.0 | 0 | 0.0 | 0 | 0.0 | 0 | 0.0 | 1 | 2.2 | 0 | 0.0 | 1 | 0.3 | 0.2 |
| polimixine | 0 | 0.0 | 0 | 0.0 | 1 | 2.5 | 0 | 0.0 | 0 | 0.0 | 0 | 0.0 | 0 | 0.0 | 0 | 0.0 | 0 | 0.0 | 1 | 0.3 | 0.5 |

B)

| **Type of molecules** | **2008 (N=37)** | **%** | **2009 (N=35)** | **%** | **2010 (N=39)** | **%** | **2011 (N=42)** | **%** | **2012 (N=36)** | **%** | **2013 (N=28)** | **%** | **2014 (N=38)** | **%** | **2015 (N=33)** | **%** | **2016 (N=38)** | **%** | **Total (N=326)** | **%** | **P-value for**  **trend** |
| --- | --- | --- | --- | --- | --- | --- | --- | --- | --- | --- | --- | --- | --- | --- | --- | --- | --- | --- | --- | --- | --- |
| carbapenems | 9 | 24.3 | 10 | 28.6 | 14 | 35.9 | 16 | 38.1 | 14 | 38.9 | 8 | 28.6 | 17 | 44.7 | 12 | 36.4 | 14 | 36.8 | 114 | 35.0 | 0.2 |
| glycopeptide | 12 | 32.4 | 9 | 25.7 | 11 | 28.2 | 19 | 45.2 | 9 | 25.0 | 7 | 25.0 | 12 | 31.6 | 14 | 42.4 | 10 | 26.3 | 103 | 31.6 | 0.9 |
| aminoglycosides | 9 | 24.3 | 11 | 31.4 | 9 | 23.1 | 15 | 35.7 | 15 | 41.7 | 4 | 14.3 | 9 | 23.7 | 9 | 27.3 | 10 | 26.3 | 91 | 27.9 | 0.7 |
| third-generation cephalosporins | 8 | 21.6 | 7 | 20.0 | 12 | 30.8 | 5 | 11.9 | 8 | 22.2 | 5 | 17.9 | 8 | 21.1 | 4 | 12.1 | 6 | 15.8 | 63 | 19.3 | 0.2 |
| penicillins and β-lactams inhibitors | 5 | 13.5 | 5 | 14.3 | 2 | 5.1 | 4 | 9.5 | 5 | 13.9 | 1 | 3.6 | 4 | 10.5 | 3 | 9.1 | 10 | 26.3 | 39 | 12.0 | 0.3 |
| fluoroquinolones | 6 | 16.2 | 4 | 11.4 | 4 | 10.3 | 4 | 9.5 | 2 | 5.6 | 5 | 17.9 | 4 | 10.5 | 2 | 6.1 | 1 | 2.6 | 32 | 9.8 | 0.09 |
| imidazole derivatives | 0 | 0.0 | 2 | 5.7 | 1 | 2.6 | 0 | 0.0 | 1 | 2.8 | 3 | 10.7 | 5 | 13.2 | 4 | 12.1 | 1 | 2.6 | 17 | 5.2 | 0.04 |
| combinations of sulfonamides and trimethoprim | 1 | 2.7 | 0 | 0.0 | 1 | 2.6 | 1 | 2.4 | 2 | 5.6 | 1 | 3.6 | 1 | 2.6 | 2 | 6.1 | 1 | 2.6 | 10 | 3.1 | 0.4 |
| macrolides | 0 | 0.0 | 1 | 2.9 | 3 | 7.7 | 1 | 2.4 | 0 | 0.0 | 2 | 7.1 | 0 | 0.0 | 0 | 0.0 | 1 | 2.6 | 8 | 2.5 | 0.6 |
| penicillins with extended spectrum | 1 | 2.7 | 0 | 0.0 | 0 | 0.0 | 1 | 2.4 | 1 | 2.8 | 2 | 7.1 | 0 | 0.0 | 1 | 3.0 | 0 | 0.0 | 6 | 1.8 | 0.9 |
| polimixine | 0 | 0.0 | 0 | 0.0 | 1 | 2.6 | 0 | 0.0 | 1 | 2.8 | 0 | 0.0 | 2 | 5.3 | 1 | 3.0 | 0 | 0.0 | 5 | 1.5 | 0.4 |
| tetraciclyne | 0 | 0.0 | 0 | 0.0 | 0 | 0.0 | 0 | 0.0 | 0 | 0.0 | 0 | 0.0 | 0 | 0.0 | 2 | 6.1 | 3 | 7.9 | 5 | 1.5 | 0.002 |
| fourth-generation cephalosporins | 0 | 0.0 | 1 | 2.9 | 0 | 0.0 | 0 | 0.0 | 0 | 0.0 | 0 | 0.0 | 0 | 0.0 | 0 | 0.0 | 0 | 0.0 | 1 | 0.3 | 0.3 |
| other antibiotics | 0 | 0.0 | 0 | 0.0 | 0 | 0.0 | 0 | 0.0 | 0 | 0.0 | 0 | 0.0 | 0 | 0.0 | 1 | 3.0 | 0 | 0.0 | 1 | 0.3 | 0.2 |
| second-generation cephalosporins | 0 | 0.0 | 0 | 0.0 | 0 | 0.0 | 1 | 2.4 | 0 | 0.0 | 0 | 0.0 | 0 | 0.0 | 0 | 0.0 | 0 | 0.0 | 1 | 0.3 | 0.7 |

C)

| **Type of molecules** | **2008 (N=52)** | **%** | **2009 (N=35)** | **%** | **2010 (N=52)** | **%** | **2011 (N=30)** | **%** | **2012 (N=46)** | **%** | **2013 (N=47)** | **%** | **2014 (N=26)** | **%** | **2015 (N=39)** | **%** | **2016 (N=39)** | **%** | **Total (N=366)** | **%** | **P-value for trend** |
| --- | --- | --- | --- | --- | --- | --- | --- | --- | --- | --- | --- | --- | --- | --- | --- | --- | --- | --- | --- | --- | --- |
| third-generation cephalosporins | 28 | 53.8 | 23 | 65.7 | 26 | 50.0 | 12 | 40.0 | 15 | 32.6 | 15 | 31.9 | 4 | 15.4 | 5 | 12.8 | 5 | 12.8 | 133 | 36.3 | <0.001 |
| first-generation cephalosporins | 3 | 5.8 | 0 | 0.0 | 8 | 15.4 | 11 | 36.7 | 18 | 39.1 | 17 | 36.2 | 12 | 46.2 | 16 | 41.0 | 18 | 46.2 | 103 | 28.1 | <0.001 |
| aminoglycosides | 11 | 21.2 | 4 | 11.4 | 8 | 15.4 | 4 | 13.3 | 8 | 17.4 | 8 | 17.0 | 3 | 11.5 | 10 | 25.6 | 8 | 20.5 | 64 | 17.5 | 0.5 |
| penicillins and β-lactams inhibitors | 8 | 15.4 | 10 | 28.6 | 6 | 11.5 | 2 | 6.7 | 4 | 8.7 | 7 | 14.9 | 3 | 11.5 | 6 | 15.4 | 3 | 7.7 | 49 | 13.4 | 0.2 |
| imidazole derivatives | 4 | 7.7 | 0 | 0.0 | 5 | 9.6 | 4 | 13.3 | 4 | 8.7 | 4 | 8.5 | 1 | 3.8 | 1 | 2.6 | 4 | 10.3 | 27 | 7.4 | 0.9 |
| second-generation cephalosporins | 1 | 1.9 | 0 | 0.0 | 2 | 3.8 | 0 | 0.0 | 2 | 4.3 | 3 | 6.4 | 6 | 23.1 | 5 | 12.8 | 8 | 20.5 | 27 | 7.4 | <0.001 |
| penicillins with extended spectrum | 3 | 5.8 | 3 | 8.6 | 1 | 1.9 | 3 | 10.0 | 1 | 2.2 | 6 | 12.8 | 0 | 0.0 | 5 | 12.8 | 1 | 2.6 | 23 | 6.3 | 0.8 |
| glycopeptide | 7 | 13.5 | 2 | 5.7 | 1 | 1.9 | 0 | 0.0 | 2 | 4.3 | 0 | 0.0 | 0 | 0.0 | 0 | 0.0 | 10 | 25.6 | 22 | 6.0 | 0.5 |
| carbapenems | 0 | 0.0 | 1 | 2.9 | 2 | 3.8 | 0 | 0.0 | 2 | 4.3 | 0 | 0.0 | 1 | 3.8 | 1 | 2.6 | 4 | 10.3 | 11 | 3.0 | 0.06 |
| combinations of sulfonamides and trimethoprim | 1 | 1.9 | 1 | 2.9 | 1 | 1.9 | 1 | 3.3 | 2 | 4.3 | 1 | 2.1 | 0 | 0.0 | 1 | 2.6 | 0 | 0.0 | 8 | 2.2 | 0.6 |
| fluoroquinolones | 0 | 0.0 | 0 | 0.0 | 3 | 5.8 | 0 | 0.0 | 0 | 0.0 | 1 | 2.1 | 0 | 0.0 | 1 | 2.6 | 1 | 2.6 | 6 | 1.6 | 0.6 |
| macrolides | 2 | 3.8 | 1 | 2.9 | 1 | 1.9 | 0 | 0.0 | 1 | 2.2 | 1 | 2.1 | 0 | 0.0 | 0 | 0.0 | 0 | 0.0 | 6 | 1.6 | 0.09 |
| β-lactams resistant penicillins | 0 | 0.0 | 0 | 0.0 | 1 | 1.9 | 0 | 0.0 | 2 | 4.3 | 0 | 0.0 | 0 | 0.0 | 0 | 0.0 | 0 | 0.0 | 3 | 0.8 | 0.8 |
| polimixine | 0 | 0.0 | 0 | 0.0 | 1 | 1.9 | 0 | 0.0 | 0 | 0.0 | 0 | 0.0 | 0 | 0.0 | 0 | 0.0 | 0 | 0.0 | 1 | 0.3 | 0.5 |
| β-lactams sensitive penicillins | 1 | 1.9 | 0 | 0.0 | 0 | 0.0 | 0 | 0.0 | 0 | 0.0 | 0 | 0.0 | 0 | 0.0 | 0 | 0.0 | 0 | 0.0 | 1 | 0.3 | 0.1 |

D)

| **Type of molecules** | **2008 (N=23)** | **%** | **2009 (N=26)** | **%** | **2010 (N=56)** | **%** | **2011 (N=39)** | **%** | **2012 (N=65)** | **%** | **2013 (N=61)** | **%** | **2014 (N=81)** | **%** | **2015 (N=84)** | **%** | **2016 (N=80)** | **%** | **Total (N=515)** | **%** | **P-value for**  **trend** |
| --- | --- | --- | --- | --- | --- | --- | --- | --- | --- | --- | --- | --- | --- | --- | --- | --- | --- | --- | --- | --- | --- |
| combinations of sulfonamides and trimethoprim | 10 | 43.5 | 4 | 15.4 | 13 | 23.2 | 21 | 53.8 | 33 | 50.8 | 32 | 52.5 | 24 | 29.6 | 41 | 48.8 | 39 | 48.8 | 217 | 42.1 | 0.02 |
| penicillins and β-lactams inhibitors | 3 | 13.0 | 5 | 19.2 | 10 | 17.9 | 2 | 5.1 | 19 | 29.2 | 29 | 47.5 | 24 | 29.6 | 10 | 11.9 | 35 | 43.8 | 137 | 26.6 | 0.002 |
| third-generation cephalosporins | 4 | 17.4 | 7 | 26.9 | 22 | 39.3 | 13 | 33.3 | 12 | 18.5 | 6 | 9.8 | 13 | 16.0 | 10 | 11.9 | 5 | 6.3 | 92 | 17.9 | <0.001 |
| fluoroquinolones | 0 | 0.0 | 2 | 7.7 | 17 | 30.4 | 9 | 23.1 | 17 | 26.2 | 21 | 34.4 | 21 | 25.9 | 1 | 1.2 | 3 | 3.8 | 91 | 17.7 | 0.007 |
| aminoglycosides | 3 | 13.0 | 5 | 19.2 | 4 | 7.1 | 3 | 7.7 | 7 | 10.8 | 13 | 21.3 | 7 | 8.6 | 7 | 8.3 | 10 | 12.5 | 59 | 11.5 | 0.8 |
| carbapenems | 1 | 4.3 | 0 | 0.0 | 3 | 5.4 | 2 | 5.1 | 8 | 12.3 | 12 | 19.7 | 7 | 8.6 | 6 | 7.1 | 7 | 8.8 | 46 | 8.9 | 0.2 |
| glycopeptide | 4 | 17.4 | 3 | 11.5 | 4 | 7.1 | 1 | 2.6 | 7 | 10.8 | 10 | 16.4 | 5 | 6.2 | 1 | 1.2 | 8 | 10.0 | 43 | 8.3 | 0.2 |
| penicillins with extended spectrum | 3 | 13.0 | 4 | 15.4 | 2 | 3.6 | 1 | 2.6 | 9 | 13.8 | 2 | 3.3 | 12 | 14.8 | 24 | 28.6 | 11 | 13.8 | 68 | 13.2 | 0.005 |
| imidazole derivatives | 0 | 0.0 | 2 | 7.7 | 8 | 14.3 | 4 | 10.3 | 2 | 3.1 | 10 | 16.4 | 4 | 4.9 | 10 | 11.9 | 6 | 7.5 | 46 | 8.9 | 0.8 |
| macrolides | 2 | 8.7 | 1 | 3.8 | 6 | 10.7 | 1 | 2.6 | 3 | 4.6 | 3 | 4.9 | 7 | 8.6 | 4 | 4.8 | 0 | 0.0 | 27 | 5.2 | 0.09 |
| first-generation cephalosporins | 0 | 0.0 | 0 | 0.0 | 1 | 1.8 | 0 | 0.0 | 1 | 1.5 | 0 | 0.0 | 1 | 1.2 | 8 | 9.5 | 1 | 1.3 | 12 | 2.3 | 0.04 |
| polimixine | 1 | 4.3 | 0 | 0.0 | 0 | 0.0 | 0 | 0.0 | 0 | 0.0 | 0 | 0.0 | 2 | 2.5 | 0 | 0.0 | 0 | 0.0 | 3 | 0.6 | 0.5 |
| tetraciclyne | 0 | 0.0 | 0 | 0.0 | 0 | 0.0 | 0 | 0.0 | 0 | 0.0 | 0 | 0.0 | 1 | 1.2 | 0 | 0.0 | 1 | 1.3 | 2 | 0.4 | 0.2 |
| β-lactams sensitive penicillins | 0 | 0.0 | 0 | 0.0 | 0 | 0.0 | 0 | 0.0 | 0 | 0.0 | 0 | 0.0 | 0 | 0.0 | 0 | 0.0 | 2 | 2.5 | 2 | 0.4 | 0.06 |
| second-generation cephalosporins | 0 | 0.0 | 0 | 0.0 | 0 | 0.0 | 0 | 0.0 | 0 | 0.0 | 0 | 0.0 | 1 | 1.2 | 0 | 0.0 | 0 | 0.0 | 1 | 0.2 | 0.6 |
